# Development of Pure Silica CHA Membranes for CO_2_ Separation

**DOI:** 10.3390/membranes11120926

**Published:** 2021-11-25

**Authors:** Gabriel Gama da Silva Figueiredo, Daishi Takayama, Katsunori Ishii, Mikihiro Nomura, Takamasa Onoki, Takuya Okuno, Hiromasa Tawarayama, Shinji Ishikawa

**Affiliations:** 1 Shibaura Institute of Technology, 3-7-5 Toyosu, Koto-ku, Tokyo 135-8548, Japan; mc20501@shibaura-it.ac.jp (G.G.d.S.F.); mc19008@shibaura-it.ac.jp (D.T.); na19101@shibaura-it.ac.jp (K.I.); 2 Sumitomo Electric Industries Ltd., 1 Taya-cho, Sakae-ku, Yokohama 244-8588, Japan; onoki-takamasa@sei.co.jp (T.O.); okuno-takuya@sei.co.jp (T.O.); tawarayama-hiromasa@sei-co.jp (H.T.); ishishin@sei.co.jp (S.I.)

**Keywords:** membrane separation, inorganic membrane, zeolite, pure silica CHA-type zeolite, CO_2_ separation, silica substrates

## Abstract

Thin pure-silica chabazite (Si-CHA) membranes have been synthesized by using a secondary growth method on a porous silica substrate. A CO_2_ permeance of 2.62 × 10^−6^ mol m^−2^ s^−1^ Pa^−1^ with a CO_2_/CH_4_ permeance ratio of 62 was obtained through a Si-CHA membrane crystallized for 8 h using a parent gel of H_2_O/SiO_2_ ratio of 4.6. The CO_2_ permeance through the Si-CHA membrane on a porous silica substrate was twice as high as that through the membrane synthesized on a porous alumina substrate, which displayed a similar zeolite layer thickness.

## 1. Introduction

The development of efficient and sustainable CO_2_ capture technologies is desired for several reasons. First, carbon dioxide is a common greenhouse gas found in combustion streams. In other words, its production is present in many industrial processes, and its accumulation in the atmosphere is a threat to many bio systems on the planet [1]. Additionally, CO_2_ is one of the main components of raw natural gas, and is responsible for pipeline corrosion problems during gas transportation [2]. For these reasons, separation technologies for carbon capture and storage (CCS) have been developed, such as pressure swing adsorption, amine scrubbing, and cryogenic distillation [3,4]. Among these separation techniques, membrane separation has presented itself as one of the most efficient methods, thus receiving increasing attention from the scientific community [5]. Fard et al. [6] reported that the global demand for membranes and membrane modules reached 15.6 billion USD in 2018, and is expected to grow annually by 8%.

Membranes are usually classified into 2 broad classes: polymeric and inorganic membranes. Although losing in terms of reproducibility, inorganic membranes are known for displaying superior thermal, chemical and mechanical stabilities when compared with polymeric membranes [7,8]. Therefore, inorganic membranes are preferentially applied for high temperature gas separation processes [9,10]. Among the materials used for the fabrication of inorganic membranes, zeolites excel as adsorbents due to their narrow and uniform pore size, high surface area, adjustable hydrophilicity and hydrophobicity, ion exchange capacity, and strong acidity [11]. In particular, chabazite (also known as CHA-type zeolite) has been researched for CO_2_ separation, due to its eight-membered ring pores of 0.38 nm. For that reason, since the molecular diameters of CO_2_ and CH_4_ are 0.33 nm and 0.38 nm, respectively, CHA membranes show high CO_2_/CH_4_ selectivity [12,13]. Kida et al. [14] reported that a CHA membrane synthesized on an alumina substrate without adding aluminum in the parent gel showed a CO_2_/CH_4_ selectivity of 130, with a CO_2_ permeance of 4.0 × 10^–6^ mol m^−2^ s^−1^ Pa^−1^. Yu et al. [15] synthesized an industrially relevant CHA membrane, with a length of 50 cm and membrane area of 100 cm^2^, which displayed a CO_2_ permeance of 1.6 × 10^–6^ mol m^−2^ s^−1^ Pa^−1^ and a CO_2_/CH_4_ selectivity as high as 236. Hasegawa et al. [16] prepared a high silica CHA-type zeolite membrane (Si/Al = 18) on a porous α-Al_2_O_3_ substrate with N_2_/SF_6_ and CO_2_/CH_4_ selectivities of 710 and 240, respectively. These high separation performances were explained by the low concentration or absence of aluminum in the parent gels during the synthesis of the CHA membrane. In the zeolite structure, cations are adhered to the negatively charged aluminum sites, which in turn increase the diffusion resistance of CO_2_ in the zeolite pores. Therefore, the preparation of pure silica CHA (Si-CHA) membranes is expected to increase CO_2_ separation performances. However, the highest achievable Si/Al ratio of CHA crystals in the conventional hydroxide medium is lower than 100 due to competition with other zeolites, such as ITQ-1, SSZ-23, and SSZ-24 [17]. For the parent gels that do not contain aluminum in its composition, CHA membranes have been prepared in fluoride medium, with rather low H_2_O/SiO_2_ ratios of 3–6 in order to obtain a successful crystallization [17,18,19]. Only a few research groups were able to synthesize high silica membranes with parent gels of higher H_2_O/SiO_2_ ratios. For example, Zhou et al. [20] were able to synthesize a high silica CHA zeolite membrane with a CO_2_/CH_4_ selectivity of 480 by using a fluoride and aluminum free parent gel with a H_2_O/SiO_2_ ratio of 120.

Si-CHA membranes are usually synthesized on porous alumina substrates in order to increase the mechanical strength of the thin CHA separation layers. However, alumina substrates are dissolved in CHA parent gels due to their alkalinity [21,22,23,24]. One of the solutions to overcome the aluminum dissolution of the porous ceramic substrates is to instead synthesize the membrane on a porous silica substrate without aluminum in its structure. The effects of using porous silica substrates to improve gas permeance were investigated in the preparation of MFI membranes [25,26,27]. Sugiyama et al. [26] were able to obtain a N_2_ permeance through a MFI membrane of 3.7 × 10^−6^ mol m^−2^ s^−1^ Pa^−1^ with a N_2_/SF_6_ permeance ratio of 328. The application of porous silica substrates was effective for MFI membranes.

Therefore, in this paper, Si-CHA membranes were crystallized on porous silica substrates. The effects of changing the synthesis conditions were investigated for dense Si-CHA membranes for CO_2_ separation. Among these synthesis conditions, the effect of adding seed crystals to the synthesis gel was studied as well. Seeding is one of most important parameters for zeolite synthesis [28]. Kong et al. [17] reported that the presence of seed crystals favors the formation of CHA zeolite more than the presence of the structure directing agent (SDA). Finally, the effects of synthesizing the Si-CHA membrane on a silica substrate were confirmed by comparing the permeation results with those through a membrane synthesized on an alumina substrate.

## 2. Materials and Methods

### 2.1. Synthesis of Si-CHA Crystals

The Si-CHA crystals were synthesized by hydrothermal synthesis based on the former literature [12]. N,N,N-trimethyl-1-adamantylammonium hydroxide (TMAdaOH: 25%, SACHEM) was selected as the structure directing agent (SDA), and Tetraethyl orthosilicate (TEOS: >99%, LS-2430, Shin-Etsu) as the silica precursor. Both compounds were mixed and stirred at 250 rpm overnight, followed by heating until obtaining a dry powder. Then, hydrofluoridric acid (HF: 46.0–48.0%, Wako) and distilled water were added to the dried powder to obtain the parent gel. The composition of the parent gel was SiO_2_:TMAdaOH:HF:H_2_O = 1:0.8:0.8:4.6 (mol/mol). This dried gel was transferred to a PFTE-lined autoclave, where hydrothermal synthesis was carried out at 150 °C for 24 h. The obtained CHA crystals were recovered by vacuum filtration and washed with distilled water. Then, after drying for 24 h, they were pulverized with an automatic mortar for 4 h. Finally, the CHA crystals were calcined in air at 600 °C for 15 h in order to remove the SDA.

### 2.2. Synthesis of Si-CHA Membranes

The Si-CHA zeolite membranes were synthesized on porous silica tubes provided by Sumitomo Electric Industries, Ltd. (Yokohama, Japan) (outer diameter: 10 mm, inner diameter: 6 mm, average pore size: 500 nm, length 30 mm). The silica substrates were coated with the Si-CHA crystals by the dip-coating method, with a Si-CHA seed crystal slurry of 8 g L^−1^ concentration and pH 2. The composition of the parent gel for the membrane synthesis was SiO_2_:TMAdaOH:HF:H_2_O = 1:0.8:0.8:3.8~5.4 (mol/mol). Moreover, 10 μm Si-CHA crystals were also added to the synthesis gel at varying quantities (0~0.25 wt%). Then, 28 g of the parent gel was smeared onto a seeded substrate, followed by hydrothermal synthesis at 150 °C for 4 h to 16 h in a Teflon-lined autoclave. After the synthesis, calcination was performed in air at 600 °C for 5 h.

In order to investigate the effects of the substrates, an alumina substrate (outer diameter: 12 mm, inner diameter: 8 mm, average pore size: 500 nm, length 30 mm, Noritake Co. (Nagoya, Japan)) was employed. The synthesis procedures were the same as those for the silica substrate.

### 2.3. Characterization

The obtained membranes were characterized by using an X-ray diffractometer (Rigaku (Tokyo, Japan)) from 5 to 40° for CuKα radiation. The morphologies of the obtained crystals and membranes were observed using a VE-8800 scanning electron microscope (SEM, KEYENCE (Osaka, Japan)). The permeation performances were measured by single gas permeance tests using the probe gases H_2_, CO_2_, N_2_, CH_4_ and SF_6_ at room temperature. As typically done, the membrane was inserted in a stainless steel module and sealed with silicone O-rings. The selected gas was fed on the outer side of the membrane with a feed flow of 200 mL/min and, after permeating the membrane, flowed to a handmade bubble flowmeter, where the volumetric flow rate and, consequently, gas permeance were determined.

## 3. Results and Discussion

### 3.1. Effects of Synthesis Time

**Figure 1:**
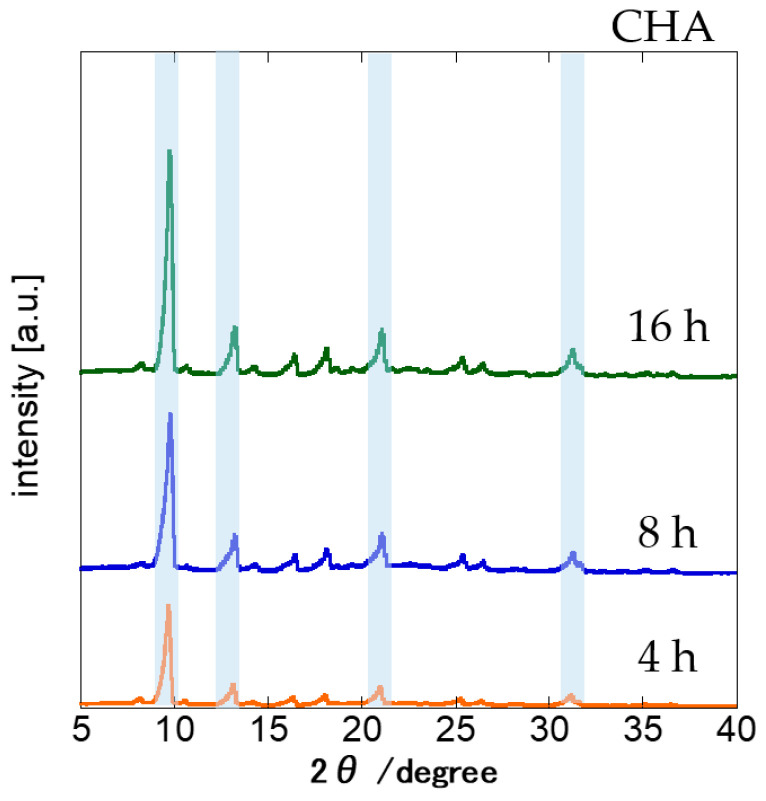
XRD measurements for the membranes crystallized for varying synthesis times (1 SiO_2_:0.8 TMAdaOH:0.8 HF:4.6 H_2_O, 0.01 wt% CHA seeds, at 423 K).

The synthesis time for the CHA zeolite membrane synthesis on the silica substrates was investigated from 4 h to 16 h at 150 °C. The H_2_O/SiO_2_ ratio and the added seed crystals were fixed at 4.6 and 0.01 wt%, respectively. Figure 1 shows the XRD patterns for the membranes. The highest diffraction peak at 9.4° refers to the (100) plane of the CHA and is considered its characteristic peak. All the other diffraction peaks were assigned for the CHA structure, with the exception of a very small peak at 8.1°. This peak was found in all membranes and refers to the (020) plane of the STT-type zeolite, a common impurity in CHA-type zeolite synthesis [18]. The obtained XRD peaks show that, starting from a synthesis time of 4 h, practically pure CHA layers were obtained, with the membrane synthesized for 16 h displaying, among the obtained membranes, the highest ratio of intensity of CHA and STT characteristic peaks. Specifically, a CHA (9.4°)/STT (8.1°) of 25.3 was obtained, which is equivalent to about 96% Si-CHA purity. CHA (9.4°)/STT (8.1°) ratios of 18 and 24 were obtained for the 4 h and 8 h synthesis, respectively. This shows that Si-CHA zeolite purity is proportional to synthesis time. The characteristic peak intensities at 9.4° increased by increasing the synthesis time. Therefore, the crystals’ sizes are also proportional to the synthesis time.

**Figure 2:**
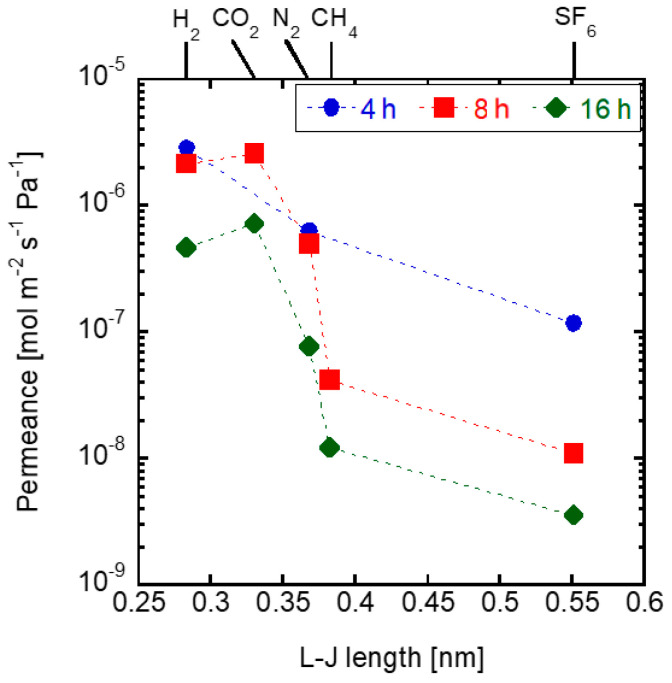
Single gas permeances through the membranes crystallized for varying synthesis times. (1 SiO_2_:0.8 TMAdaOH:0.8 HF:4.6 H_2_O, 0.01 wt% CHA seeds, at 423 K).

Figure 2 shows the single gas permeances through the obtained membranes. The H_2_/SF_6_ permeance ratio through the membrane synthesized for 4 h was 24.26. The Knudsen diffusion ratio of H_2_/SF_6_ is 8.5, showing that the membrane synthesized for 4 h was not dense enough. H_2_/SF_6_ permeance ratio through the membrane synthesized for 8 h was 196, which is much higher than that for 4 h synthesis. Furthermore, the membrane’s CO_2_ permeance was of 2.62 × 10^−6^ mol m^−2^ s^−1^ Pa^−1^, with a CO_2_/CH_4_ permeance ratio of 62. The membrane’s high selectivity can be explained by the molecular sieve mechanism due to the pore size of the CHA structure. The high CO_2_/CH_4_ permeance ratio indicates that the membrane synthesized for 8 h was dense. The membrane synthesized for 16 h displayed a similar CO_2_/CH_4_ permeance ratio of 60. Therefore, this membrane was also dense. However, its CO_2_ permeance was only 7.32 × 10^−7^ mol m^−2^ s^−1^ Pa^−1^, which is 71.2% lower than that through the 8 h synthesis membrane. The lower CO_2_ permeance can be explained by the thicker CHA layer after the 16 h synthesis. A similar result was obtained by Chew et al. [29], who synthesized on α-alumina a membrane of SAPO-34, a type of zeolite with CHA structure, which displayed an ideal CO_2_/CH_4_ selectivity of 56.

### 3.2. Effect of H_2_O/SiO_2_ Ratio of the Parent Gel

The effect of the H_2_O/SiO_2_ ratio of the parent gel was investigated. The H_2_O/SiO_2_ ratio varied from 3.8 to 5.4. The synthesis time was fixed at 16 h. The parent gel displayed a paste-like state. The viscosity of the parent gel is an important parameter, as the adherence of the paste to the surface of the substrate is necessary for the successful uniform synthesis of the zeolite layer.

**Figure 3:**
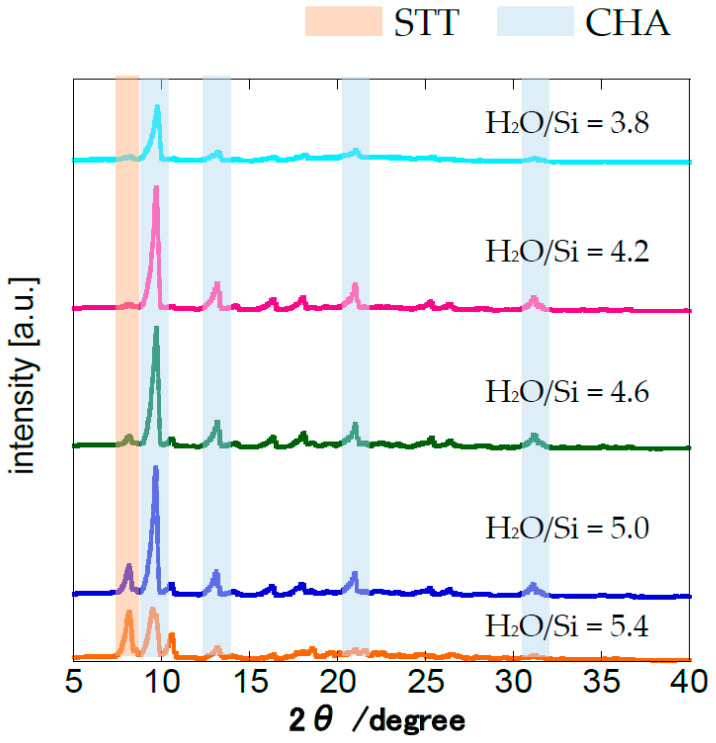
XRD measurements for the membranes crystallized with different H_2_O/SiO_2_ ratios. (1 SiO_2_:0.8 TMAdaOH:0.8 HF:X H_2_O, 0.01 wt% CHA seeds, 16 h at 423 K).

Figure 3 shows the XRD patterns for the membranes crystallized with different H_2_O/SiO_2_ ratios. The intensities at 8.1° increased when increasing the H_2_O/SiO_2_ ratio of the parent gel. Miyamoto et al. [18] reported that synthesis gels with low H_2_O/SiO_2_ ratios tend to initiate the formation of zeolites with lower framework densities. The framework densities of CHA and STT are 15.4 and 17.0 Si/nm^3^, respectively. Thus, the STT structure was more present in the surface of the membranes synthesized by the parent gels of lower silica concentration. A CHA (9.4°)/STT (8.1°) intensity ratio of 29.5 was obtained when H_2_O/SiO_2_ = 4.2, the highest calculated from these XRD measurements. The CHA (9.4°)/STT (8.1°) intensity ratio was only 13.8 when H_2_O/SiO_2_ = 3.8 due to the low viscosity of the parent gel. Therefore, the gel’s optimal H_2_O/SiO_2_ ratio is 4.2, as it tends to produce a pure CHA layer, as well as displaying good viscosity.

**Figure 4:**
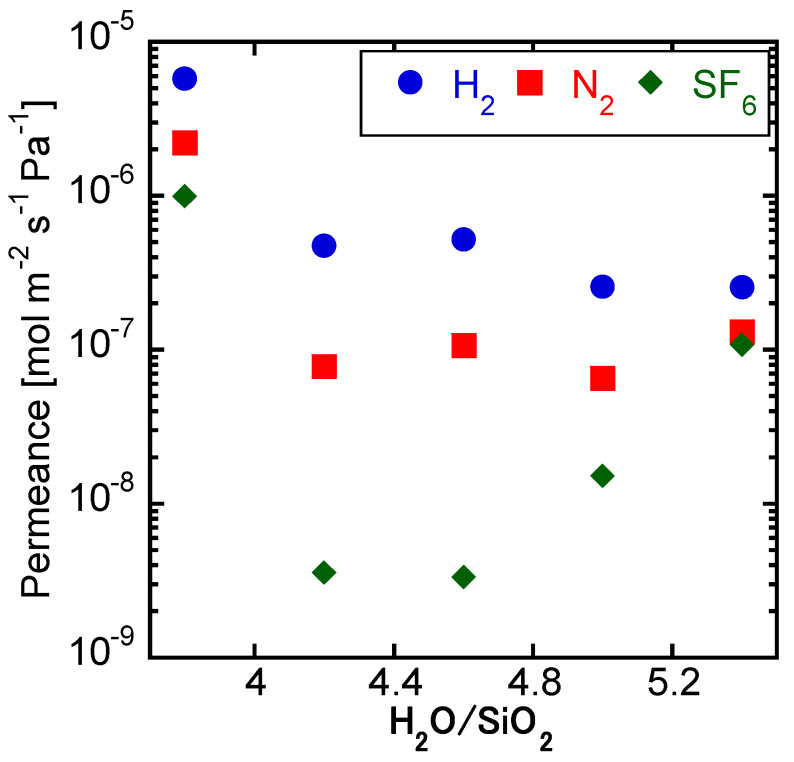
Single gas permeances through the membranes crystallized with varying H_2_O/SiO_2_. (1 SiO_2_:0.8 TMAdaOH:0.8 HF:X H_2_O, 0.01 wt% CHA seeds, 16 h at 423 K).

Figure 4 shows the single gas permeances through the membranes synthesized with different H_2_O/SiO_2_ ratios. The overall high permeance of the membrane obtained with a parent gel of H_2_O/SiO_2_ ratio of 3.8 was due to the low membrane thickness, consequent to the high viscosity of the parent gel. For this membrane, a low H_2_/SF_6_ permeation ratio of 5.81 was obtained, due to the non-uniform coating of the parent gel on the substrate prior to crystallization. On the other hand, the SF_6_ permeance increased drastically when increasing the H_2_O/SiO_2_ ratio to 5.0 and over. As a result, high H_2_/SF_6_ permeance ratios over 130 were obtained only by the membranes with H_2_O/SiO_2_ = 4.2 and 4.6. These membranes showed the highest CHA (9.4°)/STT (8.1°) peak intensity ratios as well, displaying values of 29.5 and 25.3 for the membranes synthesized with the parent gels of H_2_O/SiO_2_ ratios of 4.2 and 4.6, respectively. In order to obtain a uniform crystal layer, the STT phase in the CHA structure is not desirable.

### 3.3. Effect of Adding Seed Crystals to the Synthesis Gel

**Figure 5:**
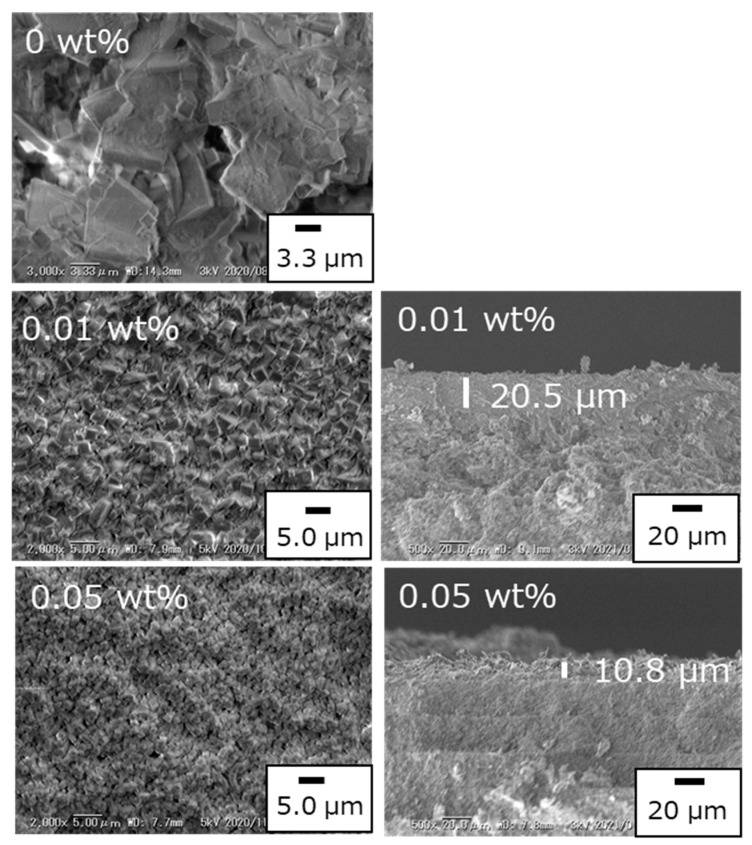
The effect on the morphology of pure silica CHA zeolite membranes by adding seed crystals (1 SiO_2_:0.8 TMAdaOH:0.8 HF:4.6 H_2_O, x wt% CHA seeds, 16 h at 423 K).

The amount of seed crystals in a parent gel of H_2_O/SiO_2_ = 4.6 was investigated in a 16 h synthesis at 150 °C. In these syntheses, the weight percentage of CHA seed crystals in the parent gel was varied from 0 to 0.25 wt%. Figure 5 shows the surface and the crosssectional images of the SEM observation of the membranes. No CHA layer was observed in the case of the parent gel without any seed crystals. On the other hand, a polycrystalline structure was found in the membranes with 0.01 wt% and 0.05 wt% seed crystals. The surface crystal size was of 1.07 μm when 0.01 wt% seed crystals were added, while a smaller crystal size of 530 nm was obtained when 0.05 wt% seed crystals were added. Since the seed crystals in the parent gel function as crystallization nuclei, the number of CHA crystals should increase by increasing the amount of CHA seed crystals added to the parent gel [30,31]. Therefore, smaller crystals are obtained with higher amounts of seed crystals, since the total amount of zeolite is limited by the total amount of coated parent gel.

**Figure 6:**
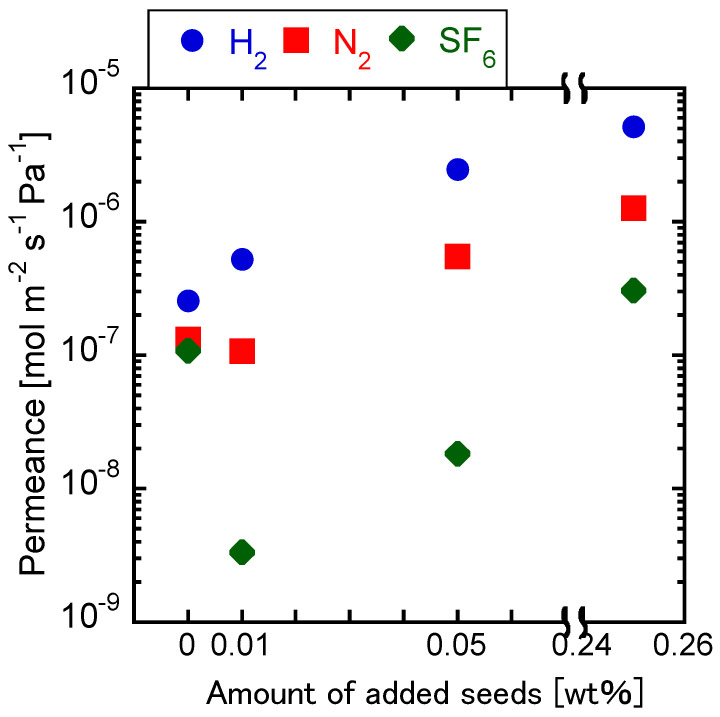
Single gas permeances through the membranes crystallized with varying seed crystal concentrations. (1 SiO_2_:0.8 TMAdaOH:0.8 HF:4.6 H_2_O, x wt% CHA seeds, 16 h at 423 K).

Figure 6 shows the single gas permeances through the membranes synthesized with different concentrations of seed crystals in the parent gel. The H_2_/SF_6_ permeance ratio was of 2.37 in the case of the membrane synthesized with no addition of seed crystals. Additionally, H_2_ permeance was lower than that through the substrate. Judging by the low H_2_/SF_6_ permeance ratio, it can be concluded that the membrane was not dense, as the molecular size of SF_6_ is larger than the pore size of CHA. However, both H_2_/SF_6_ permeance ratio and H_2_ permeance increased with increasing the amounts of seed crystals in the parent gel. As discussed before, the crystal size decreased with increasing the amount of seed crystals in the parent gel, resulting in a thinner and denser CHA layer. However, the H_2_/SF_6_ permeance ratio through the membrane prepared with the high seed crystal ratio of 0.25 wt% in the parent gel was only 16.88, lower than that through the membrane prepared with a seed crystal ratio of 0.05 wt%. The CHA layer must have been too thin to function as a dense membrane.

### 3.4. Effects of the Substrates

**Figure 7:**
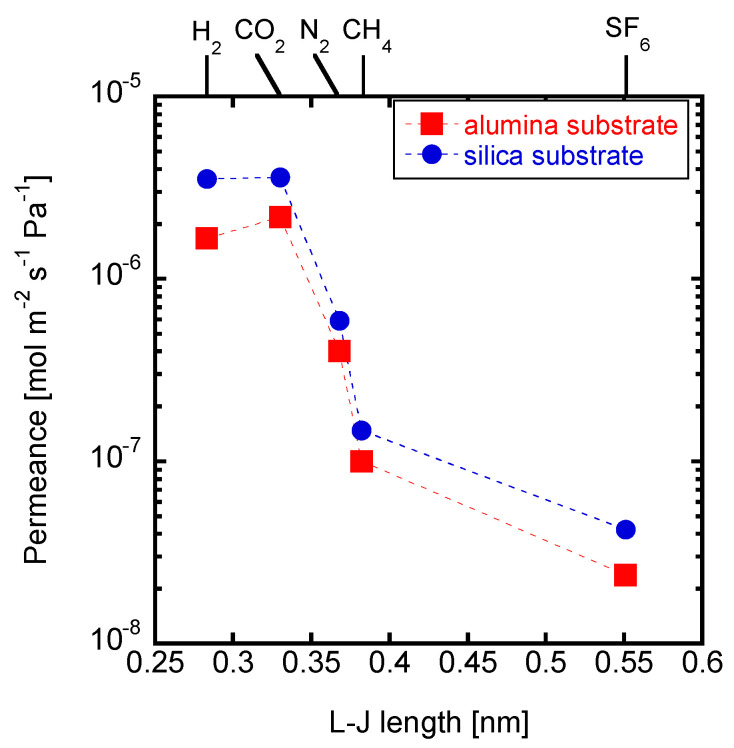
Single gas permeances through the membranes crystallized on different types of substrates (1 SiO_2_:0.8 TMAdaOH:0.8 HF:4.6 H_2_O, 0.01 wt% CHA seeds, 16 h at 423 K).

The synthesis procedure of the Si-CHA membrane on silica substrate was optimized in the former sections. In this section, a Si-CHA zeolite membrane was synthesized on an alumina substrate to confirm, by comparison, the effects of synthesizing a Si-CHA membrane on a silica substrate. Figure 7 shows the single gas permeances through the membranes synthesized on different types of substrates. Both membranes show similar H_2_/SF_6_ permeance ratios of 71.05 (alumina) and 83.80 (silica). However, the H_2_ permeance through the membrane on the silica substrate was of 3.53 × 10^−6^ mol m^−2^ s^−1^ Pa^−1^, which is about twice as high than that through the membrane on the alumina substrate. Aluminum is dissolved from the alumina substrate during CHA synthesis, and the dissolved Al from the substrates can affect the Si/Al ratio of the CHA on the alumina substrate [26]. The membrane synthesized on alumina substrate has displayed a Si/Al ratio of about 5 by an X-ray spectroscopy (EDS) analysis. Cations are found around the Al atoms in the pores of the zeolite structure, and display a form of diffusion resistance [32]. However, this is not the case when synthesizing a zeolite membrane on a porous silica substrate. Therefore, CHA membranes with a Si/Al ratio of infinite can be synthesized on silica substrates to improve permeance.

**Figure 8:**
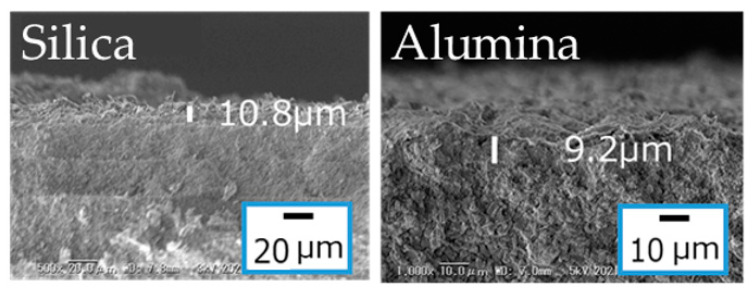
Thicknesses of CHA zeolite membranes synthesized on different porous substrates. (1 SiO_2_:0.8 TMAdaOH:0.8 HF:4.6 H_2_O, x wt% CHA seeds, 16 h at 423 K).

Figure 8 shows the CHA zeolite layer thicknesses of the membranes synthesized on porous silica and alumina substrates. Both membranes displayed similar thicknesses of 10.8 μm and 9.2 μm for the membranes synthesized on silica and alumina substrates, respectively. The membrane synthesized on the alumina substrate was obtained with 170 °C and 70 h, whereas the one synthesized on the silica substrate was obtained with 150 °C and 16 h. Therefore, apart from the fact that Si-CHA membranes synthesized on porous silica substrates are more permeable than CHA membranes synthesized on alumina substrates, silica substrates also display a possibility of synthesizing CHA membranes on them with milder synthesis conditions.

Finally, both membranes have shown CO_2_/CH_4_ separation potential, displaying CO_2_/CH_4_ separation factors over 20.

## 4. Conclusions

Si-CHA zeolite membranes were synthesized on novel silica substrates for the first time. The synthesis procedures were optimized to obtain a CO_2_/CH_4_ selective membrane.

The membrane has displayed an increase of the separation layer thickness by increasing the synthesis time. CO_2_/CH_4_ selectivity was slightly lower in the 16 h synthesis (59) when compared to the membrane synthesized for 8 h (62). However, H_2_ permeation was 71.2% lower in the case of the 16 h synthesis.

The synthesis gel should display an ideal concentration of water. If the water concentration is too low (H_2_O/SiO_2_ < 3.8), the gel becomes too viscous. As a result, a zeolite layer displaying poor uniformity is obtained. On the other hand, a too high water concentration (H_2_O/SiO_2_ > 5) results in the synthesis of STT zeolite. In this study, a water concentration of H_2_O/SiO_2_ = 4.2 has resulted in the best uniformity of the zeolite layer among the studied range of H_2_O/SiO_2_ ratio.

The concentration of Si-CHA seed crystals in the synthesis gel was investigated for the first time in this paper. The most prominent effect observed when adding seed crystals to the synthesis gel was the increase of the zeolite layer’s density. The increased number of nuclei must have resulted in the synthesis of smaller Si-CHA crystals. The H_2_/SF_6_ ideal selectivity was increased when 0.01–0.05 wt% of seed crystals were added to the synthesis gel.

Finally, regarding the choice of substrate, a clear effect in the overall gas permeance of the membranes was observed. It was possible to obtain an overall permeance twice as high as the same membrane synthesized on a porous alumina substrate by synthesizing the Si-CHA membrane on a silica porous substrate instead. By avoiding the effect of alumina dissolution from the substrate, pore blockage effect by ions and water can be averted.

The membrane with the highest CO_2_/CH_4_ separation potential was synthesized on a porous silica substrate with the following conditions: H_2_O/SiO_2_ ratio of 4.6 and addition of 0.01 wt% Si-CHA seed crystals in an 8 h synthesis at 150 °C. It is important to note that the synthesis of Si-CHA type zeolite membrane on porous silica substrates is still a novel technique, and still has much room for improvement.

## Data Availability

Not applicable.

## References

[1] Whitfield M. (1974). Accumulation of fossil CO_2_ in the atmosphere and in the sea. Nature.

[2] Rufford T.E., Smart S., Watson G.C.Y., Graham B.F., Boxall J., Diniz da Costa J.C., May E.F. (2012). The removal of CO_2_ and N_2_ from natural gas: A review of conventional and emerging process technologies. J. Pet. Sci. Eng..

[3] Behrooz H.A., Hoseini M., Mahamadzade M., Ranjbaran N. Economic Comparison between Membrane and Adsorption Processes for Separation of CO_2_ and CH_4_ Mixture. Proceedings of the 5th National Conference on New Researches in Chemistry and Chemical Engineering.

[4] Belaissaoui B., Moullec Y.L., Willson D., Favre E. (2012). Hybrid membrane cryogenic process for post-combustion CO_2_ capture. J. Memb. Sci..

[5] Brunetti A., Macedonio F., Barbieri G., Drioli E. (2015). Membrane engineering for environmental protection and sustainable industrial growth: Options for water and gas treatment. Environ. Eng. Res..

[6] Fard A.K., McKay G., Buekenhoudt A., Sulaiti H.A., Motmans F., Khraisheh M., Atieh M. (2018). Inorganic Membranes: Preparation and Application for Water Treatment and Desalination. Materials.

[7] Benfer S., Popp U., Richter H., Siewert C., Tomandl G. (2001). Development and characterization of ceramic nanofiltration membranes. Sep. Purif. Technol..

[8] Nomura M., Yamaguchi T., Nakao S. (1997). Silicalite Membranes Modified by Counter diffusion CVD Technique. Ind. Eng. Chem. Res..

[9] van Veen H.M., van Delft Y.C., Engelen C.W.R., Pex P.P.A.C. (2001). Dewatering of organics by pervaporation with silica membranes. Sep. Purif. Technol..

[10] Songolzadeh M., Soleimani M., Ravanchi M.T., Songolzadeh R. (2014). Carbon Dioxide Separation from Flue Gases: A Technological Review Emphasizing Reduction in Greenhouse Gas Emissions. Sci. World J..

[11] Miyamoto M., Fujioka Y., Yogo K. (2012). Pure silica CHA type zeolite for CO2 separation using pressure swing adsorption at high pressure. J. Mater. Chem..

[12] Kida K., Maeta Y., Yogo K. (2017). Preparation and gas permeation properties on pure silica CHA-type zeolite membranes. J. Memb. Sci..

[13] Zhou H., Kean W. (2018). The ‘ideal selectivity’ vs. ‘true selectivity’ for permeation of gas mixtures in nanoporous membranes. IOP Conference Series: Materials Science and Engineering.

[14] Kida K., Maeta Y., Yogo K. (2018). Pure silica CHA-type zeolite membranes for dry and humidified CO_2_/CH_4_ mixtures separation. Sep. Purif. Technol..

[15] Yu L., Nobandegani M.S., Hedlund J. (2022). Industrially relevant CHA membranes for CO_2_/CH_4_ separation. J. Memb. Sci..

[16] Hasegawa Y., Abe C., Natsui M., Ikeda A. (2021). Gas Permeation Properties of High-Silica CHA-Type Membrane. Membranes.

[17] Kong X., Qiu H., Zhang Y., Tang X., Meng D., Yang S., Guo W., Xu N., Kong L., Zhang Y. (2021). Seeded synthesis of all-silica CHA zeolites in diluted mother liquor. Micro. Meso. Mater..

[18] Miyamoto M., Nakatani T., Fujioka Y., Yogo K. (2015). Verified synthesis of pure silica CHA-type zeolite in fluoride media. Micro. Meso. Mater..

[19] Kim E., Cai W., Baik H., Choi J. (2013). Uniform Si-CHA Zeolite Layers Formed by a Selective Sonication-Assisted Deposition Method. Angew. Chem. Int. Ed..

[20] Zhou J., Gao F., Sun K., Jin X., Zhang Y., Liu B., Zhou R., Benfer S., Popp U., Richter H. (2020). Green Synthesis of highly CO_2_-selective CHA zeolite membranes in all-silica and fluoride-free solution for CO_2_/CH_4_ separations. Energy Fuels.

[21] Zarubin D.P., Nemkina N.V. (1990). The solubility of amorphous silica in an alkaline aqueous medium at a constant ionic strength. Russ. J. Inorg. Chem..

[22] Franke M.D., Ernst W.R., Myerson A.S. (1987). Kinetics of dissolution of alumina in acidic solution. Am. Inst. Chem. Eng..

[23] Sano T., Yanagishita H., Kiyozumi Y., Mizukami F., Haraya K. (1994). Separation of ethanol/water mixture by silicalite membrane on pervaporation. J. Memb. Sci..

[24] Eilertsen E.A., Milsen M.H., Wendelbo R., Olsbye U., Lillerud K.P. Synthesis of high silica CHA zeolites with controlled Si/Al ratio. Proceedings of the 4th International FEZA Conference.

[25] Imasaka S., Nakai A., Araki S., Yamamoto H. (2018). Synthesis and Gas Permeation of STT-type Zeolite Membranes. J. Jpn. Pet. Inst..

[26] Sugiyama Y., Ikarugi S., Oura K., Ikeda A., Matsuyama E., Ono R., Tawarayama H., Saito T., Kuwahara K., Nomura M. (2015). MFI zeolite membranes prepared on novel silica substrates. J. Chem. Eng. Jpn..

[27] Tanizume S., Maehara S., Ishii K., Onoki T., Okuno T., Tawarayama H., Ishikawa S., Nomura M. (2020). Reaction of methanol to olefin using a membrane contactor on a silica substrate. Sep. Purif. Tech..

[28] Oleksiak M.D., Rimer J.D. (2014). Synthesis of zeolites in the absence of organic structure-directing agents: Factors governing crystal selection and polymorphism. Rev. Chem. Eng..

[29] Chew T.L., Ahmad A.L. (2016). Gas Permeation Properties of Modified SAPO-34 Zeolite Membranes. Procedia Eng..

[30] Grand J., Awala H., Mintova S. (2016). Mechanism of zeolites crystal growth: New findings and open questions. Cryst. Eng. Comm..

[31] Nanev C.N. (2020). Relationship between number and sizes of crystals growing in batchcrystallization: Nuclei number density, nucleation kinetics and crystal polydispersity. J. Cryst. Growth.

[32] Drummond D., De Jonge A., Rees L.V.C. (1983). Ion exchange kinetics in zeolite A. J. Phys. Chem..

